# The Ward Bathroom in Design and Detail

**Published:** 1912-04-20

**Authors:** 


### THE MAKING OF A MODERN HOSPITAL.*

### The Ward Bathroom in Design and Detail.

The ward bathroom is contained in the annexes, and its position will, of course, vary with the arrangement of these. In the older hospitals it was very often placed either at the end of a long corridor, some distance away from the ward, or in the immediate neighbourhood of the more public annexes, such as the hall or ward kitchen. The former position was supremely inconvenient by reason of the fact that in order to reach the bathroom the patients had to traverse a long, usually badly heated, and invariably draughty corridor; the latter position was undesirable because it robbed the bathroom of that amount of privacy which is essential in this portion of the annexes. It is not always easy, however, so to arrange the bathroom that the essentials of convenient and easy—and, be it added, safe—access and absolute privacy are secured.

### Lessons from German Hospitals.

In the plans of newer hospitals various ingenious attempts have been made to secure these essentials. In most of the newer German hospitals the bathroom is placed at the extreme end of the ward, and is entered either directly from the ward or, more usually, by a narrow central corridor, which is well warmed. At the Virchow, for instance, double-end bathrooms are provided, approached by a short corridor which opens at one end into the ward, while the other entrance forms the lateral entrance to the ward; the consequence of this arrangement is that the corridor is with difficulty kept warm, especially as the flooring is of polished tiles. At the new academic clinics at Heidelberg the ward bathrooms are simply arranged at the end of each ward, the administrative annexes being, as at the Virchow, centrally placed; this arrangement, with no corridor, but simply a door of entry into the ward itself, is also followed at several new hospitals in America and in Italy. The new institutions in France and Belgium seem to prefer the central arrangement, the designers arguing that this renders the bathroom easier of access, and gives better administrative control. In most English institutions the bathrooms are arranged at the extreme end of the wards; this ensures a certain amount of privacy but requires the connecting corridors to be well heated, and is said to be less convenient for administrative purposes. In the model ward unit approved by the Belgian Hospital Commission the central arrangement is favoured.

A preliminary consideration is the size of the bathroom. In other words, how many baths ought to be provided for a ward? The generally accepted rule is that there ought to be a bath for every ten beds; the German and French authorities allow a bath for every fifteen beds on an average; the newer American hospitals are more generous, and provide a bath for every eight patients, or even for every five. In certain newer Continental hospitals this rule is disregarded, and the bathing accommodation is utterly inadequate. At the Nuremberg Hospital, for example, there is a bath for every thirty-five patients; in some of the wards at the Baltimore Johns Hopkins Hospital a bath is provided for every twenty-five beds; in the surgical clinic of the new Rome Hospital a bath is allowed for every twenty beds; while in the new obstetrical clinic at Vienna there is a double bathroom for a ward of twenty-five patients. According to Kuhn, a ward bathroom for a twenty-bedded ward should be of not less than 20 square mètres in area, and in such a room two to three baths can be installed. For obvious reasons it is better to have a separate room for each bath, or, where that is impossible, to have the baths partitioned off so as to ensure privacy. In any case it is desirable that the ward bathroom should be a bathroom, and not a lavatory. The lavatory accommodation should be distinct, so that the bathroom can be used at any time during the day or night without interfering with the convenience of patients who require the washing lavatories.

It must be further remembered that the ward bathrooms are not meant to serve as small balneo-therapeutic departments. In cases of emergency they may be used as such, but ordinarily therapeutic baths and balneological methods of treatment are to be carried out in the balneo-therapeutic department attached to the institution of which the ward is a unit. The sole exception to this rule is in the case of the surgical wards, where "permanent water-beds" are often used in cases of burns. Such permanent baths should be arranged in a separate room, for the installation demands special precautions and special apparatus. In English hospitals permanent bath-beds of such a character are generally improvised in the ward itself, the water being led into and out of the bath by a double series of rubber tubes, while a temporary bathroom is improvised by the use of screens. In the newer hospitals abroad the surgical wards contain provision for at least one permanent water-bed in a special room which is distinct from the ward bathroom, and which generally corresponds to the additional isolation-room on the medical side. In our hospitals, even of the newest type, no provision has been made for the installation of such permanent water-beds—a point which hospital architects may with advantage consider in the future.

### The Bathroom Walls and Floor.

The walls and floor of the bathrooms should be covered with some smooth, non-absorbing, impermeable material; the floor preferably of mosaic or tiles: the slipperiness and coldness are guarded against by the use of movable cork matting and felt or cork-soled slippers, which should be supplied to every person using the bathroom. Tiled walls and floors are very suitable, and if some white or neutral colour is chosen the use of this material greatly improves the appearance of the bathroom. Linoleum

* Previous articles have appeared on Oct. 7, 14, 21, 28, Nov. 4, 18, Dec. 9, 30, Feb. 10, March 16, and April 6.

and wood flooring cannot be considered so suitable; if the former is used it must be frequently renewed. Here, as elsewhere in the unit, all corners must be rounded and the interior of the room rendered as smooth surfaced as possible, so as to permit of easy and complete cleansing by means of a hose. Special attention must be paid to the ceiling. In some bathrooms an overhead window or skylight is provided; this is undesirable for many reasons. The ceiling should be plain, preferably slightly concave, and painted or enamelled in some impermeable, non-absorbing, permanent, washable paint. Many new hospitals have their bathrooms tiled from floor to ceiling; the tiling is carried right round the room and on to the ceiling itself, a plinth being thus avoided and regularity obtained. This is the ideal method, but it is expensive and scarcely necessary where a good washable paint can be used for the ceiling.

### Heating and Ventilation.

Great attention should be paid to the ventilation and heating of the bathroom—both points which are apt to be regarded as of secondary importance by the architect. Lighting is of less consequence; a good double window admitting a sufficiency of light is all that is required; it is not necessary that every corner of the bathroom should be flooded with direct sunlight. The window panes are best frosted on the outside for a distance of a couple of feet; the use of corrugated plate-glass windows must be warned against, as they serve to lodge dust and dirt and generally cut off far more light than plain frosted panes. Adequate artificial lighting must be provided for by electric glow-lamps, well out of reach of the bather or of water splashing, though they must be so arranged that they serve to illuminate the bath itself. Gas illumination is undesirable. The main problem is to provide adequate heating and yet to ensure that the room should be properly ventilated. This is perhaps best secured by open ventilators fixed close to the floor behind small radiators; the current of air passing through is warmed by the radiator and is evenly distributed through the room without creating a draught. An open fire is very cheerful and sometimes very convenient in a bathroom, especially for warming towels, dressing-gowns, &c., but it is generally found impracticable to have one, except in cases where there is only one bathroom. A gas fire is convenient and easy to manipulate but is not desirable for various reasons.

We now come to the furnishing of the bathroom. Here the keynote should be simplicity. The only essential piece of furniture is the bath itself. This should be in one piece, and should be so placed that it allows of free access from all sides. It must, therefore, not be too close to the wall, but an ample space must be provided all round to enable the floor to be swept and cleaned. Either movable or fixed baths may be used; both have their advantages and disadvantages. The fixed bath is generally popular, but the movable bath is becoming daily more widely employed. If a good first-class model is chosen the movable bath is as stable and as easily kept clean as the fixed, and its great advantage—namely, that it can be transferred from place to place should any necessity arise, is a decided point in its favour. In the case of a movable bath the castors or wheels should be strong, simple in construction, and noiseless; they are preferably made of metal, enamelled; rubber castors very soon get useless. So far as fixed baths are concerned, these may either be fixed to the floor itself, or slightly raised above it. The former method is the better, since it makes the bath easier to get into by the patients. In some cases the bath has actually been built into the floor, forming an integral part of it, as was the case in some of the old Roman baths, the rim being then almost flush with the floor. Such a bath is very convenient and comfortable, but not at all easy to keep clean, and very difficult to repair when broken or damaged.

### A Type to Avoid.

No matter which type is selected, the bath should be of first-class material. The old type of enamelled zinc bath is most undesirable, and has been given up in modern bathrooms. In its stead there is a wide choice between the enamelled copper, nickelled steel (both very expensive types), plated aluminium (a type which, however well it wears, always looks third rate), enamelled iron, the extravagant manganese bronze (which with the pure marble bath only the ambitious faddist class of institution can afford), and the generally useful glazed china and earthenware baths. The last type is the most suitable for institutional use so far as fixed baths are concerned; for movable baths the enamelled copper bath is the best, owing to its lightness and its capacity for resisting hard wear. The enamelled iron bath, enamelled in leadless porcelain glaze enamel, is a very good type, but has the objection that it is difficult to repair; it is the type which has been adopted in the public baths at Berlin, Vienna, and Rome. The nickel-plated steel type is very easily cleaned and always looks well; baths of this type have been in constant and hard use at the Urban hospital since 1890, and when our Commissioner saw them in 1908 were still in excellent condition. They are, however, an expensive model compared with the china type. Whatever type is adopted, the bath should be in one piece, with no joints, sharp corners, or angles; its surface, whether glazed or enamelled, should be smooth like well-polished ivory, and its covering should be permanent. Care must be taken, therefore, that the glaze or enamel is of a make that has been well tested and that is not liable to crack or scale. Wood or composition baths are quite out of place in a modern bathroom. The size of the bath depends largely on individual taste. Most institutional baths are made too deep, so that many patients find it a matter of some difficulty to get into them; a fairly broad and comparatively shallow model is very convenient. The model preferred on the Continent is generally 2 feet 4 inches deep.

### Taps and Pipes.

The water supplied to the bath should be brought by fairly wide tubes provided with easily managed taps for both hot and cold water. It is preferable that the tubes should not be encased in the wall. The taps must be of a type to be manipulated by the fingers, not, as is sometimes seen, of the type that requires a screw. A shower bath for both hot or cold should be fixed over each bath; the rose of this should be almost flush with the ceiling, and the outflow should be managed and regulated by a screw or push tap, placed where it can easily be reached by the bather himself. This douche arrangement is a great convenience and is installed with but little extra cost and trouble; it should be provided in every ward bathroom, not as a separate piece of mechanism, but as part of the bath itself. Another point on which we would lay stress is the necessity for providing a fairly powerful call-bell, the push of which must be so placed that it can be manipulated by anyone while lying in the bath. Many serious accidents have happened owing to neglect of this very elementary precaution. Where the bell-push is placed at the door, some distance away from the bath itself, it is almost useless, or even worse than useless, for it gives a false sense of security. The danger is that a convalescent patient, while in the bath, may be attacked by sudden faintness, which may prevent him from getting out of the bath and calling for assistance, although he may still be able to ring a bell placed within reach of him as he lies in the bath.

The rest of the bathroom furniture may be described in a few words. A towel rack or stand, a cork mat, a fire screen where an open fireplace is present, a movable rack for soap, scrubbing brush, or sponge—it being understood that everyone who uses the bath brings his or her own soap, sponge, and brush—and a clock are all that are required. The rack is best made of enamelled ironware and placed on the rim of the bath, where it can slide forwards or backwards to suit the bather's convenience. The cork mats must be dry, and need considerable care to keep them in order. An easy chair or lounge, upholstered in American leather or washable imitation leather, is also a piece of furniture that may justifiably be added. Yet an additional item is a wall thermometer to regulate the temperature of the room; the bath thermometer should be suspended in the bath itself and should be in charge of the nurse or sister of the ward.

The ward bathroom, it will be seen, is an important portion of the annexes of the ward unit, and as such it demands the careful consideration of the hospital architect and superintendent. A good bathroom is a great convenience to all the patients, who should be encouraged to make use of it as much as possible, since cleanliness in a hospital counts for much more than it does outside. A badly arranged and indifferently equipped bathroom, on the other hand, very often becomes a source of trouble and worry, if not of actual danger, to everyone connected with the ward.

